# LAT1 supports mitotic progression through Golgi unlinking in an amino acid transport activity-independent manner

**DOI:** 10.1016/j.jbc.2024.107761

**Published:** 2024-09-11

**Authors:** Sakura Yanagida, Ryuzaburo Yuki, Youhei Saito, Yuji Nakayama

**Affiliations:** Laboratory of Biochemistry and Molecular Biology, Kyoto Pharmaceutical University, Kyoto, Japan

**Keywords:** LAT1, amino acid transport, mitosis, spindle orientation, Golgi, Golgi unlinking, centrosome, mitotic spindle

## Abstract

Amino acid transporters play a vital role in cellular homeostasis by maintaining protein synthesis. L-type amino acid transporter 1 (LAT1/SLC7A5/CD98lc) is a major transporter of large neutral amino acids in cancer cells because of its predominant expression. Although amino acid restriction with various amino acid analog treatments is known to induce mitotic defects, the involvement of amino acid transporters in cell division remains unclear. In this study, we identified that LAT1 is responsible for mitotic progression in a transport activity-independent manner. LAT1 knockdown activates the spindle assembly checkpoint, leading to a delay in metaphase. LAT1 maintains proper spindle orientation with confinement of the lateral cortex localization of the NuMA protein, which mediates the pulling force against the mitotic spindle toward the lateral cortex. Unexpectedly, JPH203, an inhibitor of LAT1 amino acid transport activity, does not affect mitotic progression. Moreover, the transport activity-deficient LAT1 mutant maintains the proper spindle orientation and mitotic progression. LAT1 forms a heterodimer with CD98 (SLC3A2/CD98hc) both in interphase and mitosis. Although CD98 knockdown decreases the plasma membrane localization of LAT1, it does not affect mitotic progression. LAT1 is localized to the Golgi and ER not only at the plasma membrane in interphase, and promotes Golgi unlinking during the mitotic entry, leading to centrosome maturation. These results suggest that LAT1 supports mitotic progression in an amino acid transport activity-independent manner and that Golgi-localized LAT1 is important for mitotic progression through the acceleration of Golgi unlinking and centrosome maturation. These findings reveal a novel LAT1 function in mitosis.

Mitotic cell division is an important process for conveying genetic information to daughter cells, and centrosomes play crucial roles in this process (1). From the G2 to mitotic entry, the pericentriolar materials are gradually recruited to the pericentrioles, which increases the centrosome size (2). During this centrosome maturation process, mitotic kinases, such as Aurora A and PLK1, are recruited to the centrosomes, where Aurora A promotes the maturation process (3). After nuclear envelope breakdown, the mature centrosomes assemble mitotic spindle microtubules, and the mitotic spindle is oriented properly parallel to the substratum dependently on Aurora A (4). Spindle microtubules capture the chromosomes and align them at the spindle equator. To accomplish faithful chromosome segregation, the spindle assembly checkpoint (SAC) ensures the proper attachment of microtubules to kinetochores *via* sensing the tension at the kinetochores in metaphase cells (5). Once the SAC is satisfied, the sister chromatids can separate (6). Persistent SAC activation results in mitotic cell death; therefore, various anti-mitotic drugs have been clinically used and are being developed these days (7).

During the mitotic entry, various organelles change their structures for proper mitotic progression and equal segregation (8). The structural changes in the Golgi ribbon support centrosome maturation and subsequent spindle organization (9, 10, 11). The Golgi ribbon is generated by interconnecting Golgi stacks during the G1/S phase, and is unlinked into Golgi stacks again during the late G2 phase and prophase, followed by unstacking into cisternae and successive vesiculation. JNK and MAPK signaling promotes Golgi unlinking and thereby subsequent G2/M transition (12, 13). Moreover, the Golgi unlinking-mediated activation of Src promotes Aurora A recruitment to the centrosomes, thus leading to centrosome maturation (14). Prevention of Golgi disassembly by artificial linking between Golgi stacks disrupts spindle formation and results in mitotic arrest (15); therefore, proper structural changes of the Golgi are important for cell division.

L-type amino acid transporter 1 (LAT1/SLC7A5/CD98lc) is a transporter of large neutral amino acids, such as Val, Leu, and Met (16). LAT1 forms a heterodimer with CD98 (SLC3A2/CD98hc) and is trafficked from the perinuclear region to the plasma membrane (17, 18). LAT1 is highly expressed in various cancer cells and supports homeostasis by maintaining protein synthesis (19). Therefore, LAT1 knockdown or the inhibition of its transport activity by LAT1 inhibitors, such as JPH203 and OKY-034, suppresses the proliferation of various cancers (20, 21). Since the reduction of intracellular amino acids by treatment with various amino acid analogs has been reported to induce mitotic defects (22, 23), LAT1 inhibition is presumed to induce various mitotic defects or block mitotic entry *via* insufficient protein synthesis; however, this effect has not been investigated. LAT1 inhibitors have been in clinical trials in several cancers (24). The combination of LAT1 inhibitors and anti-mitotic drugs may be an efficient cancer therapy *via* the induction of mitotic cell death.

In this study, we demonstrate that LAT1 knockdown delays mitotic progression by proper SAC activation and induces spindle misorientation. Surprisingly, treatment with an inhibitor of the LAT1’s transport activity does not affect mitotic progression or spindle orientation. Consistent with this result, the re-expression of the LAT1 mutant lacking amino acid transport activity could mitigate the delay in mitotic progression and the defect in spindle orientation caused by LAT1 knockdown. This suggests that LAT1 supports spindle orientation and mitotic progression in a transport activity-independent manner. Furthermore, we found that LAT1 is localized at the Golgi region and that LAT1 knockdown represses Golgi unlinking during the mitotic entry and recruitment of Aurora A to the centrosomes. Our results highlight a novel function of the amino acid transporter.

## Results

### LAT1-mediated regulation of mitotic progression

**Figure 1:**
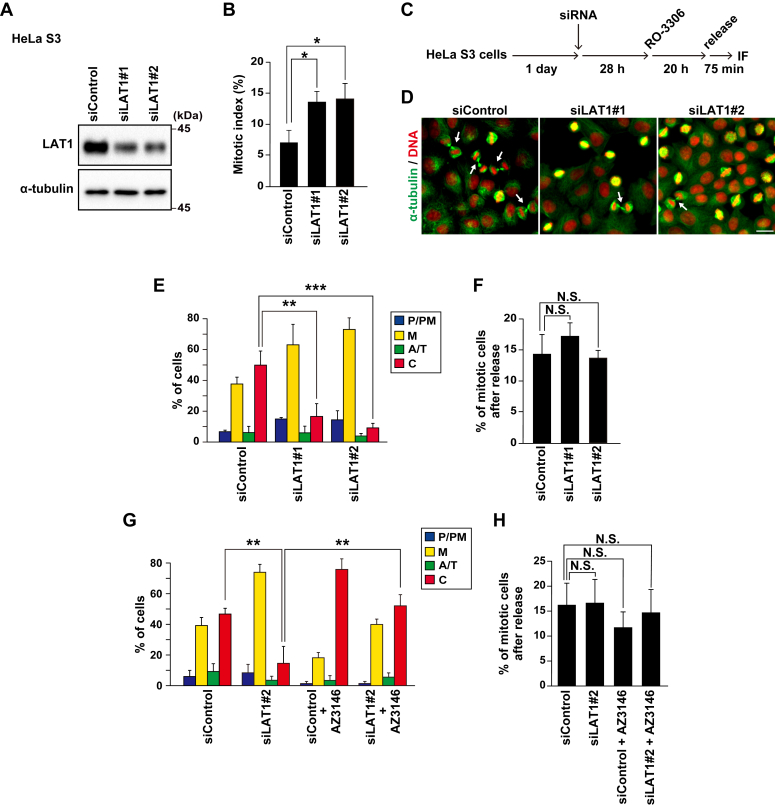
**LAT1 knockdown delays mitotic progression *via* activation of spindle assembly checkpoint**. HeLa S3 cells were transfected with control siRNA (siControl) or LAT1-targeting siRNAs (siLAT1#1 and #2). *A*, at 48 h after transfection, Western blot analysis was performed with the indicated antibodies. *B*, at 48 h after transfection, the HeLa S3 cells were fixed and stained for phospho-Histone H3 (pS10) and DNA. The mitotic indices are plotted as the mean ± SD of three independent experiments (n > 1000). Statistical analysis was performed using one-way ANOVA (*F* = 10.37, *p* = 0.011), and asterisks indicate significant differences (Dunnett’s test, ∗*p* < 0.05). *C–F*, At 28 h after siRNA transfection, the HeLa S3 cells were treated with 6 μM RO-3306 for 20 h, washed with PBS(+), and cultured for a further 75 min. The cells were then fixed and stained for α-tubulin (*green*) and DNA (*red*). *C*, a schematic depiction of the synchronization method is shown. *D*, representative images are shown. Arrows indicate the cells during cytokinesis. Scale bar, 20 μm. The mitotic cells were classified into four groups (see “Experimental procedures”). The percentages of cells of the each group (*E*) or the percentage of mitotic cells (*F*) are plotted as the mean ± SD of three independent experiments [n > 200 in panel (*E*), n > 1000 in panel (*F*)]. Statistical analysis was performed using one-way ANOVA [*F* = 25.56, *p* = 0.001 in panel (*E*); *F* = 1.936, *p* = 0.224 in panel (*F*)], and asterisks indicate significant differences (Dunnett’s test, ∗∗*p* < 0.01, ∗∗∗*p* < 0.001, N.S., not significant). *G* and *H*, At 28 h after siRNA transfection, the HeLa S3 cells were treated with 6 μM RO-3306, washed, and cultured with or without 2 μM AZ-3146 treatment for a further 75 min. The percentages of cells of the each group (*G*) or the percentage of mitotic cells (*H*) are plotted as the mean ± SD of three independent experiments [n > 200 in panel (*G*), n > 1000 in panel (*H*)]. Statistical analysis was performed using two-way ANOVA in panel (*G*) (siRNA, *F* = 43.447, *p* = 0.000; AZ3146, *F* = 55.156, *p* = 0.000; interaction, *F* = 1.473, *p* = 0.259) and in panel (*H*) (siRNA, *F* = 0.005, *p* = 0.948; AZ3146, *F* = 0.432, *p* = 0.529; interaction, *F* = 0.012, *p* = 0.917). Asterisks indicate significant differences (Tukey’s test, ∗∗*p* < 0.01, N.S., not significant).

To investigate the role of the LAT1 amino acid transporter in mitotic progression, we knocked down LAT1 expression using two different siRNAs in human cervical cancer HeLa S3 cells and confirmed LAT1 knockdown by Western blot analysis (Fig. 1*A*). LAT1 knockdown increased the mitotic index (Fig. 1*B*), suggesting a possibility that LAT1 knockdown prolonged mitotic duration; therefore, we evaluated their mitotic progression by synchronization of cells at the G2/M boundary. We previously demonstrated that mitotic progression could be monitored by quantitating the percentage of mitotic subphases after release from G2/M arrest caused by treatment with the CDK1 inhibitor RO-3306 (25). We classified the mitotic cells into four groups based on the morphologies of their DNA and microtubules: prophase and prometaphase (P/PM), metaphase (M), anaphase and telophase (A/T), and cytokinesis (C). In the control cells, approximately half of the cells progressed to cytokinesis at 75 min after release from RO-3306 synchronization (Fig. 1, *C*–*E*, siControl). In contrast, treatment with LAT1-targeting two different siRNAs strongly decreased the percentage of cytokinesis (Fig. 1, *D* and *E*, siLAT1#1 and #2), suggesting that LAT1 knockdown slows down mitotic progression. Since SAC contributes to correcting mitotic defects by causing mitotic arrest (26), we examined the involvement of SAC activation in the delay in mitotic progression caused by LAT1 knockdown. Treatment of cells with AZ3146, an Mps1 kinase inhibitor (27), mitigated the decrease in cytokinesis percentage induced by LAT1 knockdown (Fig. 1*G*). These results suggest that LAT1 knockdown delays mitotic progression through SAC activation.

The percentage of cells that entered mitosis was comparable between the control cells and the LAT1-knockdown cells (Fig. 1, *F* and *H*), indicating that LAT1 knockdown does not affect mitotic entry. Stable knockdown of LAT1 using shRNA is known to arrest the cell cycle in interphase (28). However, considering that LAT1-knockdown cells were properly synchronized at the G2/M boundary similar to the control cells, a strong arrest in interphase might not occur in cells treated with LAT1-targeting siRNAs for 2 days. Mitotic progression is likely more sensitive to LAT1 knockdown than cell cycle progression in interphase and proliferation.

### Amino acid transport activity-independent regulation of mitotic progression

**Figure 2:**
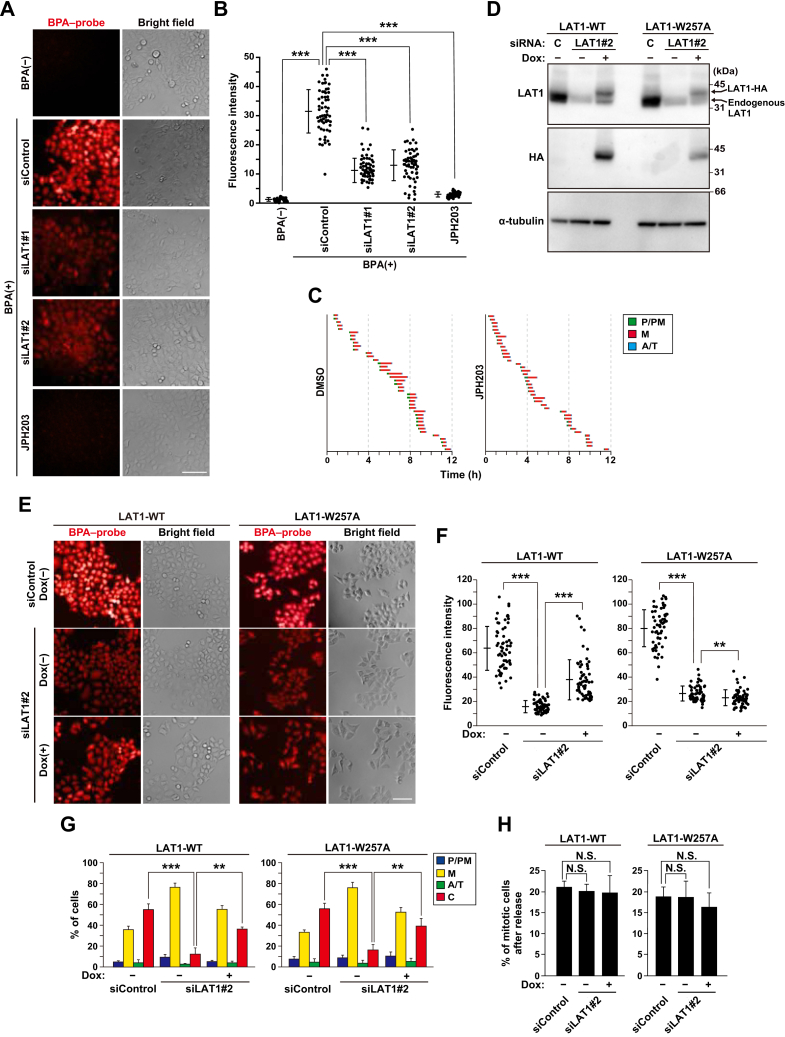
**LAT1 supports proper mitotic progression in an amino acid transport activity-independent manner**. *A* and *B*, HeLa S3 cells were transfected with control siRNA (siControl) or LAT1-targeting siRNAs (siLAT1#1 and #2), or treated with 30 μM JPH203 for 48 h. The amino acid transport ability was evaluated using boronophenylalanine (BPA) and its probe (see “Experimental procedures”). *A*, representative images are shown. Images of the BPA–probe fluorescence were pseudocolored as red. Scale bar, 100 μm. *B*, the fluorescence intensity of the BPA–probe fluorescent compound was measured and plotted as the mean ± SD from a representative experiment (n = 60). Statistical analysis was performed using Welch’s ANOVA (*F* = 413, *p* = 0.000), and asterisks indicate significant differences (Games–Howell test, ∗∗∗*p* < 0.001). *C*, HeLa S3 cells were treated with 30 μM JPH203 for 48 h, and the cells were monitored for a further 12 h by time-lapse imaging with 0.1 μM Hoechst 33342. The duration of each mitotic phase is presented: prophase/prometaphase (P/PM: chromosome condensation, *green*), metaphase (M: chromosome alignment, *red*), and anaphase/telophase (A/T: from anaphase onset to cleavage furrow ingression, *blue*). In total, 40 mitotic cells were examined. *D–F*, doxycycline (Dox)-inducible LAT1-WT or LAT1-W257A (HeLa S3/LAT1-WT or LAT1-W257A) cell lines were transfected with siControl or siLAT1#2 with or without 2 μg/ml Dox. At 48 h after transfection, Western blot analysis was performed (*D*), and the amino acid transport ability was evaluated (*E*, *F*). *E*, representative images are shown. Images of the BPA–probe fluorescence were pseudocolored as red. Scale bar, 100 μm. *F*, The fluorescence intensity of the BPA–probe fluorescent compound was measured and plotted as the mean ± SD from a representative experiment (n = 60). Statistical analysis was performed using Welch’s ANOVA (*F* = 160.7, *p* = 0.000 in the *left panel*; *F* = 576, *p* = 0.000 in the *right panel*), and asterisks indicate significant differences (Games–Howell test, ∗∗*p* < 0.01; ∗∗∗*p* < 0.001). *G and H*, HeLa S3/LAT1-WT or LAT1-W257A cells were transfected with siControl or siLAT1#2 with or without 2 μg/ml Dox treatment. At 28 h after siRNA transfection, the cells were treated with 6 μM RO-3306 for 20 h with or without Dox treatment, washed, and cultured for a further 90 min. The cells were then stained for α-tubulin and DNA. The percentages of cells of the each group (G) or the percentage of mitotic cells (H) are plotted as the mean ± SD of four independent experiments [n > 200 in panel (*G*), n > 1000 in panel (H)]. Statistical analysis was performed using one-way ANOVA in panel (*G*) (*F* = 61.12, *p* = 0.000 in the *left panel*; *F* = 33.17, *p* = 0.000 in the *right panel*) and (*H*) (*F* = 0.082, *p* = 0.922 in the *left panel*; *F* = 0.537, *p* = 0.61 in the *right panel*). Asterisks indicate significant differences (Tukey’s test, ∗∗*p* < 0.01; ∗∗∗*p* < 0.001; N.S., not significant).

LAT1 is a major transporter of large neutral amino acids in cancer cells (17); therefore, we hypothesized that a decrease in protein synthesis by LAT1 knockdown-induced shortage of intracellular amino acids might affect mitotic progression. To evaluate the LAT1-mediated amino acid uptake, we performed a fluorescence-based amino acid uptake assay (see “Experimental procedures”). Based on this assay, boronophenylalanine (BPA), an analog of phenylalanine, is transported into the cells and rapidly reacts with the cell-permeable probe, thereby generating the BPA–probe fluorescent structure in cells (29). Indeed, the intracellular blue fluorescence was clearly observed upon incubation with BPA and the probe (Fig. 2, *A* and *B*, BPA(−) vs BPA(+) siControl, pseudocolored red). As expected from previous reports (30), LAT1 knockdown decreased the fluorescence intensity (Fig. 2, *A* and *B*, BPA(+) siLAT1#1 and #2). Moreover, treatment with the LAT1 inhibitor JPH203 strongly decreased the fluorescence intensity (Fig. 2, *A* and *B*, BPA(+) JPH203), confirming the assay’s specificity for evaluating the amino acid transport activity of LAT1. Since JPH203 strongly inhibited the uptake of large neutral amino acids compared with LAT1 knockdown, we examined the effect of JPH203 treatment on mitotic progression by time-lapse imaging analysis. We distinguished the mitotic subphases based on chromosome morphology visualized with Hoechst 33342 (see “Experimental procedures”). We classified the mitotic phases into three groups: from chromosome condensation to alignment (prophase/prometaphase, P/PM), aligned chromosomes (metaphase, M), and from chromosome segregation to furrow ingression (anaphase/telophase, A/T). Unexpectedly, JPH203 treatment did not cause a delay in the mitotic progression in HeLa S3 cells (Fig. 2*C*). Similarly, mitotic defects were hardly observed in pancreatic cancer MIA PaCa-2 cells upon treatment with the IC50 dose of JPH203 (Fig. S1).

To substantiate the relevance between the role of LAT1 in mitotic progression and its amino acid transport activity, we generated an amino acid uptake-deficient mutant of LAT1 with a Trp257-to-Ala substitution (LAT1-W257A), as previously reported (31), and established doxycycline (Dox)-inducible cell lines of LAT1 wildtype or the mutant (Fig. 2*D*). Re-expression of wild-type LAT1 partly mitigated the LAT1 knockdown-caused decrease in the fluorescence intensity (Fig. 2, *E* and *F*) and cytokinesis percentage (Fig. 2, *G* and *H*). Interestingly, even though the LAT1-W257A mutant lost its amino acid transport activity (Fig. 2, *E* and *F*), LAT1-W257A increased the cytokinesis percentage compared with the LAT1 knockdown to levels comparable to the wild-type LAT1 re-expression (Fig. 2, *G* and *H*). Collectively, these results suggest that LAT1 supports mitotic progression in an amino acid transport activity-independent manner.

### Prolongation of metaphase duration by LAT1 knockdown

**Figure 3:**
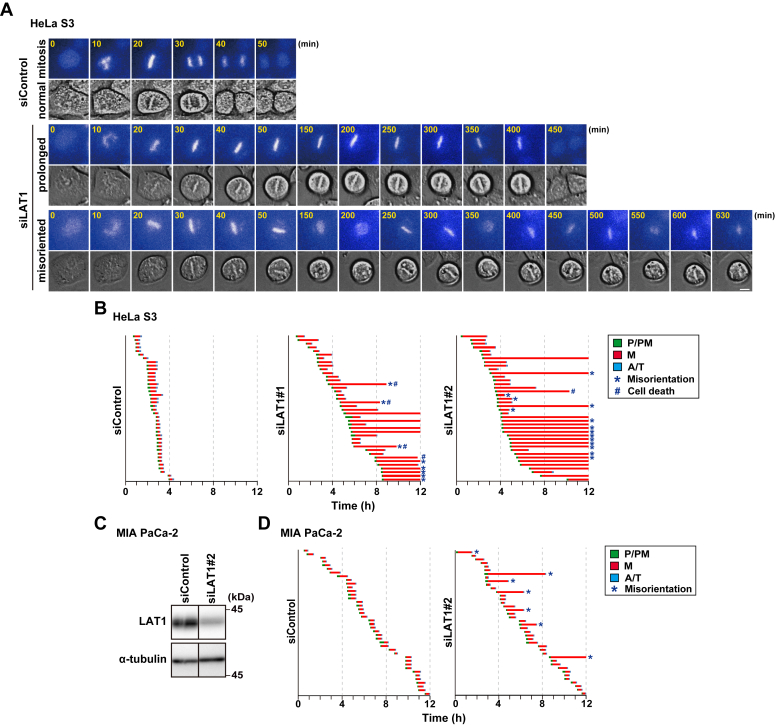
**LAT1 knockdown prolongs metaphase duration**. *A* and *B*, HeLa S3 cells were transfected with control siRNA (siControl) or LAT1-targeting siRNAs (siLAT1#1 and #2). At 19 h after siRNA transfection, the cells were treated with 4 mM thymidine for 20 h, washed with PBS(−), and cultured for a further 9 h. The cells were then monitored for 12 h by time-lapse imaging with 0.1 μM Hoechst 33342. *A*, images show the typical phenotypes of siRNA-treated mitotic cells: cells that exhibit normal mitosis (normal mitosis), the prolonged duration of metaphase (prolonged), and spindle misorientation (misorientation). Scale bar, 10 μm. *B*, the graphs are shown as indicated in Fig. 2*C*. ∗, misorientation; #, cell death. In total, 40 mitotic cells were examined. *C* and *D*, MIA PaCa-2 cells were transfected with siControl or siLAT1#2. *C*, at 48 h after transfection, Western blot analysis was performed with the indicated antibodies. *D*, at 48 h after siRNA transfection, the cells were monitored for 12 h by time-lapse imaging with 0.1 μM Hoechst 33342. The graphs are shown as indicated in panel (*B*), and *orange* bars indicate the mitotic exit without chromosome segregation (slippage).

To examine which subphases were delayed by LAT1 knockdown, we performed a time-lapse imaging analysis. Normal mitosis in the control cells was completed within approximately 1 h (Fig. 3*A*, siControl, normal mitosis). In the LAT1-knockdown cells, chromosome segregation was severely impeded after chromosome alignment (Fig. 3*A*, siLAT1). Approximately half of the LAT1-knockdown cells exhibited an extended prolongation of the metaphase duration (Fig. 3*B*). However, no effect on the duration of prophase/prometaphase or anaphase/telophase was observed. Moreover, mitotic chromosomes appeared to repeat their aligned and unaligned states in the LAT1-knockdown cells from the top view during time-lapse imaging (Fig. 3, *A* and *B*, siLAT1, misoriented). Since this result is expected to be a defect in the spindle orientation of metaphase cells, LAT1 knockdown might lead to spindle misorientation. We further examined the role of LAT1 in mitotic progression in MIA PaCa-2 cells. LAT1 knockdown also prolonged their metaphase duration and might induce defects in their spindle orientation (Fig. 3, *C* and *D*). These results suggest that LAT1 is important for SAC satisfaction during metaphase.

### Maintenance of spindle orientation by LAT1

**Figure 4:**
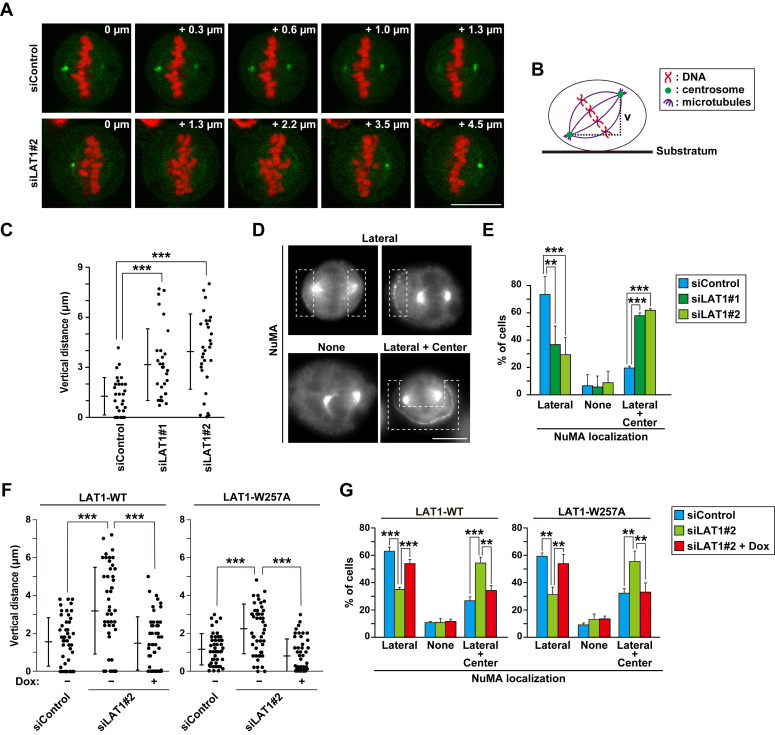
**LAT1 supports the spindle orientation with proper confinement of the lateral cortex localization of NuMA in a transport activity-independent manner**. *A–E*, HeLa S3 cells were transfected with control siRNA (siControl) or LAT1-targeting siRNAs (siLAT1#1 and #2). At 28 h after siRNA transfection, the cells were treated with 6 μM RO-3306 for 20 h, washed, and cultured for a further 60 min. *A–C*, the cells were fixed and stained for γ-tubulin (*green*) and DNA (*red*). *A*, Z-section images at 0.32-μm intervals (Z-axis) of a representative cell are shown from the lowest (0 μm) to the highest (4.5 μm) position. Scale bar, 10 μm. *B*, a schematic depiction of the metaphase cells is shown. The vertical distance (v) indicates the difference between the heights of two centrosomes within a cell. *C*, the difference in the height of γ-tubulin within a single cell was measured and plotted as the mean ± SD (n = 30). Statistical analysis was performed using Welch’s ANOVA (*F* = 15.45, *p* = 0.000), and asterisks indicate significant differences (Games–Howell test, ∗∗∗*p* < 0.001). *D* and *E*, the cells were fixed and stained for NuMA (gray) and DNA. *D*, representative images are shown. Scale bar, 10 μm. Dashed lines indicate NuMA localization at the cortex. The cells were classified into three groups (see “Experimental procedures”). *E*, the percentages of cells of each group are plotted as the mean ± SD of three independent experiments (n > 100). Statistical analysis was performed using one-way ANOVA [*F* = 32.08, *p* = 0.000 (lateral); *F* = 55.34, *p* = 0.000 (lateral + center)], and asterisks indicate significant differences (Dunnett’s test, ∗∗*p* < 0.01; ∗∗∗*p* < 0.001). *F* and *G*, HeLa S3/LAT1-WT or LAT1-W257A cells were transfected with siControl or siLAT1#2 with or without 2 μg/ml Dox. At 28 h after siRNA transfection, the cells were treated with 6 μM RO-3306 for 20 h with or without Dox, washed, and cultured for a further 60 min. The cells were then fixed and stained for γ-tubulin (*F*) or NuMA (*G*). *F*, the difference in the height of γ-tubulin within a single cell (vertical distance) was measured and plotted as the mean ± SD (n = 50). Statistical analysis was performed using Welch’s ANOVA (*F* = 16.05, *p* = 0.000 in the left panel; *F* = 26.03, *p* = 0.000 in the right panel), and asterisks indicate significant differences (Games–Howell test, ∗∗∗*p* < 0.001). *G*, the percentages of cells of each group are plotted as the mean ± SD of three independent experiments (n > 100). Statistical analysis was performed using one-way ANOVA in the left panel [*F* = 105.6, *p* = 0.000 (lateral); *F* = 47.26, *p* = 0.000 (lateral + center)] and in the right panel [*F* = 23.52, *p* = 0.001 (lateral); *F* = 14.09, *p* = 0.005 (lateral + center)]. Asterisks indicate significant differences (Tukey’s test, ∗∗*p* < 0.01; ∗∗∗*p* < 0.001).

We investigated how LAT1 supports mitotic progression. Since time-lapse imaging analysis showed that spindle orientation may be affected by LAT1 knockdown (Fig. 3), we precisely evaluated the spindle orientation by immunostaining γ-tubulin, a centrosome marker, and capturing z-stack images. Compared with the control cells, the focal points of the two centrosomes were largely different in the LAT1-knockdown cells, indicating a tilted spindle orientation (Fig. 4, *A*–*C*). Proper spindle orientation is regulated by a pulling force from the lateral side of the cell cortex (32, 33). The NuMA/LGN/Gαi force generator complex at the lateral cortex grasps the astral microtubules through the minus end-directed dynein motor complex, thereby mediating the pulling force against the mitotic spindle. Dysregulation of force generator proteins causes spindle misorientation (34, 35); therefore, we examined whether LAT1 knockdown affected NuMA protein localization. In most of the control cells, NuMA localization was mainly confined to the lateral cell cortex of the metaphase cells (Fig. 4*D*). However, LAT1 knockdown decreased the lateral localization but increased the localization to the other regions of the cortex instead (Fig. 4*D*). Plasma membrane localization of NuMA was classified into three groups (Fig. 4, *D* and *E*): one or both sides of the lateral cortex (lateral), unobserved (none), and lateral plus center cortex (lateral + center). Quantification of NuMA localization indicated a decrease in the lateral localization in LAT1-knockdown cells, suggesting that LAT1 maintains the proper localization of the NuMA protein. Finally, we examined the involvement of the amino acid transport activity of LAT1 in spindle orientation. Re-expression of both the LAT1 wildtype and W257A mutant decreased the difference in height between the two centrosomes and increased the lateral localization of NuMA (Fig. 4, *F* and *G*). Collectively, these results suggest that the transport activity-independent function of LAT1 supports spindle orientation along with the confinement of NuMA localization to the lateral cell cortex.

### Dispensable role of plasma membrane-localized LAT1 in mitotic progression

**Figure 5:**
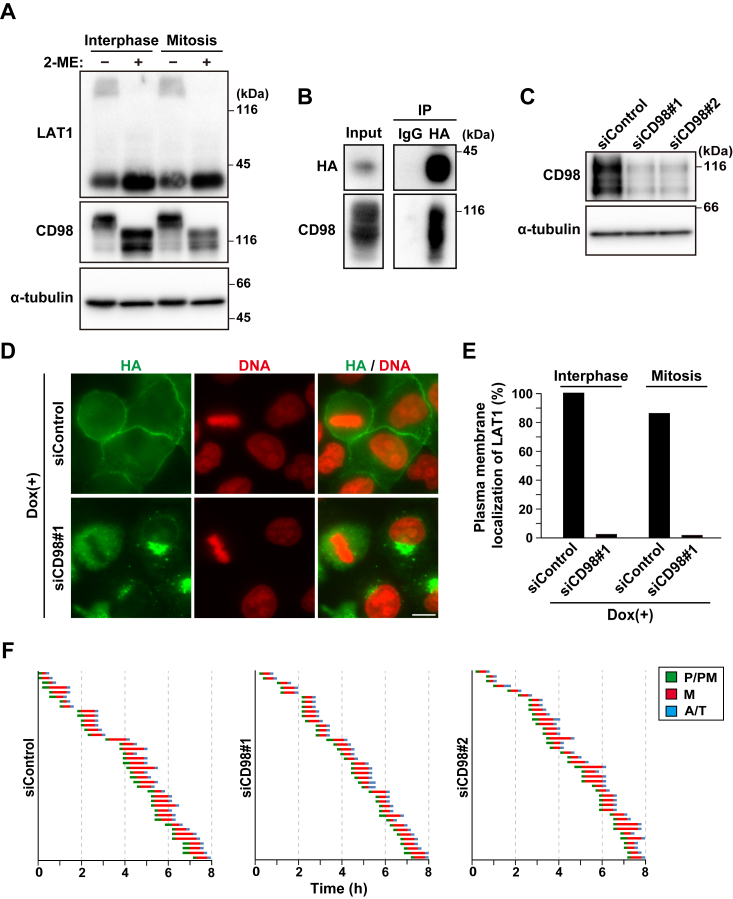
**Plasma membrane-localized LAT1 is dispensable for mitotic progression**. *A*, HeLa S3 cells were treated with 5 μM of S-Trityl-L-cysteine (STLC) for 16 h, and mitotic cells were collected by mitotic shake-off. Whole-cell lysates from asynchronous cells and mitotic cells were prepared in the presence or absence of 2-mercaptoethanol (2-ME) (see “Experimental procedures”). Western blot analysis was performed with the indicated antibodies. *B*, HeLa S3/LAT1-WT cells were treated with 2 μg/ml Dox for 32 h and then treated with 5 μM STLC for a further 16 h to induce arrest at mitosis. Mitotic cells were collected by mitotic shake-off. LAT-WT was immunoprecipitated with an anti-HA antibody in the absence of 2-ME and subjected to Western blot analysis using the indicated antibodies. *C*, HeLa S3 cells were transfected with control siRNA (siControl) or CD98-targeting siRNAs (siCD98#1 and #2). At 48 h after transfection, Western blot analysis was performed with the indicated antibodies. *D* and *E*, HeLa S3/LAT1-WT cells transfected with siControl or siCD98#1 were cultured for 48 h in the presence of 1 μg/ml Dox treatment during the last 24 h. The cells were then fixed and stained for HA (*green*) and DNA (*red*). *D*, representative images are shown. Scale bar, 10 μm. *E*, the percentage of cells exhibiting LAT1 localization at the plasma membrane is plotted (n > 50). *F*, HeLa S3 cells were transfected with siControl, siCD98#1, or siCD98#2. At 48 h after siRNA transfection, the cells were monitored for 8 h by time-lapse imaging with 0.1 μM Hoechst 33342. The graphs are shown as indicated in Fig. 2*C*.

CD98 forms a heterodimer with LAT1 and guides the trafficking of LAT1 from the perinuclear region to the plasma membrane in interphase (18), enabling LAT1 to uptake amino acids into cells. Since LAT1 regulates mitotic progression in a transport activity-independent manner (Fig. 2), it is possible that plasma membrane-localized LAT1 would be dispensable for the regulation of mitosis; therefore, we investigated it by CD98 knockdown. We first examined whether LAT1 is heterodimerized with CD98 also in mitosis. Previous studies have shown that treatment of cell lysates with the reducing agent 2-mercaptoethanol (2-ME) abolished the heterodimerization of LAT1 and CD98 (36). Indeed, the 2-ME treatment decreased the LAT1/CD98 heterodimer bands above 116 kDa in the interphase cell lysates (Fig. 5*A*, interphase). Similar results were obtained in the mitotic cell lysates (Fig. 5*A*, mitosis). Furthermore, an immunoprecipitation assay showed that Dox-induced LAT1 wild type interacted with CD98 in mitosis (Fig. 5*B*), suggesting that LAT1 heterodimerizes with CD98 both in interphase and mitosis. While LAT1 was mainly localized at the plasma membrane in interphase, CD98 knockdown greatly reduced the plasma membrane localization of LAT1 and promoted its localization at the perinuclear region, as previously reported (Fig. 5, *C*–*E*, interphase) (18). Also in mitotic cells, the plasma membrane localization of LAT1 was greatly reduced (Fig. 5, *D* and *E*, mitosis). Despite the drastic change in LAT1 localization, CD98 knockdown did not affect the duration of mitosis and spindle orientation (Fig. 5, *C* and *F*). These results suggest that the plasma membrane-localized LAT1 and its association with CD98 are dispensable for mitotic progression.

### LAT1-mediated Golgi unlinking and Aurora A recruitment to the centrosomes

**Figure 6:**
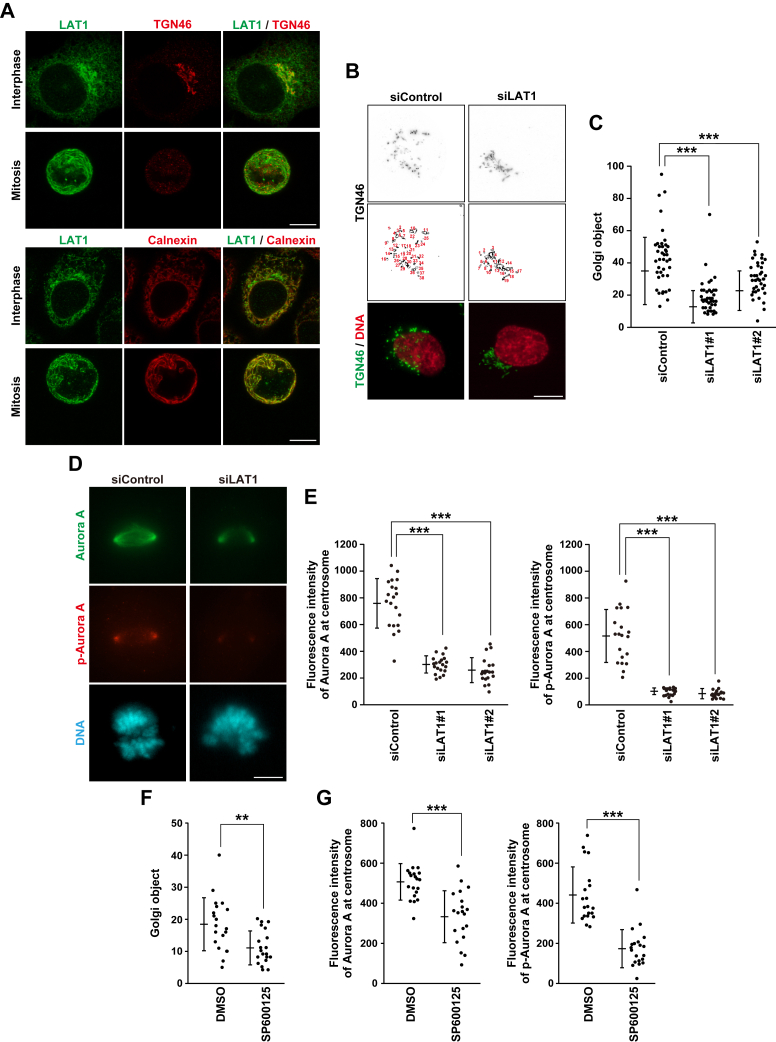
**LAT1 promotes Golgi unlinking along with Aurora A recruitment to the centrosomes**. *A*, HeLa S3 cells were fixed with formaldehyde and stained for LAT1 (*green*) and TGN46 (*red*) or calnexin (*red*). Representative images are shown. Scale bar, 10 μm. *B–E*, HeLa S3 cells were transfected with control siRNA (siControl) or LAT1-targeting siRNAs (siLAT1#1 and #2). At 19 h after siRNA transfection, the cells were treated with 4 mM thymidine for 19 h and washed with PBS(−). *B* and *C*, the cells were cultured for a further 9 h or 10 h in siControl or siLAT1 cells, respectively, to analyze the Golgi structure in prophase. The cells were fixed with MeOH and stained for TGN46 (*gray* or *green*) and DNA (*red*). *B*, representative z-stack images are shown, and Golgi objects based on TGN46 staining are numbered (see “Experimental procedures”). Scale bar, 10 μm. *C*, the number of Golgi object within a cell was measured and plotted as the mean ± SD from a representative experiment of two independent experiments (n = 40). Statistical analysis was performed using Welch’s ANOVA (*F* = 319, *p* = 0.000), and asterisks indicate significant differences (Games–Howell test, ∗∗∗*p* < 0.001). *D and E*, The cells were cultured for a further 9.5 h or 10.5 h in siControl or siLAT1 cells, respectively, to analyze Aurora A recruitment in prometaphase cells. Then, the cells were fixed with MeOH and stained for Aurora A (*green*), phospho-Aurora A (pT288) (*red*), and DNA (*cyan*). *D*, representative z-stack images are shown. Scale bar, 10 μm. *E*, the fluorescence intensity of Aurora A (*left*) or phospho-Aurora A (pT288) (*right*) at centrosomes in prometaphase per cell was measured (see “Experimental procedures”), and the average between the two centrosomes was plotted as the mean ± SD from a representative experiment of two independent experiments (n = 20). Statistical analysis was performed using Welch’s ANOVA (*F* = 99.8, *p* = 0.000 in the *left panel*; *F* = 88.6, *p* = 0.000 in the *right panel*), and asterisks indicate significant differences (Games–Howell test, ∗∗∗*p* < 0.001). *F and G*, HeLa S3 cells were treated with DMSO or 30 μM SP600125 for 2 h. *F*, the cells were fixed with MeOH and stained for TGN46 and DNA. The number of Golgi objects within a cell was measured and plotted as the mean ± SD from a representative experiment of two independent experiments (n = 20). *G*, the cells were fixed with MeOH and stained for Aurora A, phospho-Aurora A (pT288), and DNA. The fluorescence intensity of Aurora A (*left*) or phospho-Aurora A (pT288) (*right*) at centrosomes in prometaphase per cell was measured, and the average between the two centrosomes was plotted as the mean ± SD from a representative experiment of two independent experiments (n = 20). Asterisks indicate significant differences [Student’s *t* test in panel (*F* and *G*), ∗∗*p* < 0.01; ∗∗∗*p* < 0.001].

LAT1-mediated regulation of mitotic progression may occur through intracellular LAT1. Fluorescence microscopy clearly showed that endogenous LAT1 was primarily observed at the plasma membrane in MeOH-fixed cells but it was observed at the perinuclear region and the cytoplasm in formaldehyde-fixed cells (Fig. S2*A*). We doubly stained cells for LAT1 and a Golgi maker TGN46 or an endoplasmic reticulum (ER) marker calnexin. While LAT1 was colocalized with TGN46 and calnexin in formaldehyde-fixed interphase cells (Fig. 6*A*, Interphase), it was primarily colocalized with calnexin in mitotic cells (Fig. 6*A*, Mitosis). Because the Golgi ribbon is disassembled into vesicles during mitosis (11), TGN46 staining was weakened, and we could not detect the colocalization of LAT1 and TGN46. Staining of endogenous LAT1 was confirmed by LAT1 knockdown (Fig. S2*B*), suggesting that LAT1 is localized at the Golgi and ER during interphase.

Unlinking the Golgi ribbon into Golgi stacks during the mitotic entry is important for Aurora A localization to the centrosomes and subsequent centrosome maturation (14); therefore, we investigated the effect of LAT1 knockdown on the Golgi unlinking in prophase cells. The Golgi structure appeared to spread out in prophase cells compared with G1/S or early G2 cells (Fig. S2*C*), as previously reported (37). We analyzed the degree of Golgi unlinking using Image J software with “Analyse Particle” function after background subtraction and threshold application (Fig. S2*D*) (see “Experimental procedures”). In LAT1-knockdown cells, the Golgi structure was more condensed at the perinuclear region in prophase compared with control cells (Fig. 6*B*), and the measured number of “Golgi object” was clearly decreased (Fig. 6*C*). After vesiculation of the Golgi stacks in metaphase cells, LAT1 knockdown did not affect the Golgi structure (Fig. S2*E*). Moreover, LAT1 knockdown decreased the fluorescence intensity of Aurora A and phospho-Aurora A (pT288), which is the active form of Aurora A, at the centrosomes in prometaphase (Fig. 6, *D* and *E*). Furthermore, we confirmed that treatment with SP600125, an inhibitor of JNK kinase, decreased the number of “Golgi object” in prophase and the fluorescence intensity of Aurora A and phospho-Aurora A at centrosomes in prometaphase (Fig. 6, *F* and *G*), as described previously (12, 14).

In interphase, the ER tubule extends toward the cell periphery in a motor protein-dependent or -independent manner (38). Motor protein-independent ER extension is mediated by the association between the ER and the growing microtubule plus ends through the microtubule-binding protein EB1 and ER proteins, such as STIM1 and REEP3/4 (39). In mitosis, previous reports showed that phosphorylation of STIM1 dissociates the ER-microtubule association and that REEP3/4 ensures the exclusion of the ER from the mitotic spindle (40, 41). The expression of a non-phosphorylatable mutant of STIM1 or REEP3/4 knockdown resulted in an abnormal accumulation of ER on the mitotic spindle, which caused mitotic defects. We examined the effect of LAT1 knockdown on ER distribution in mitosis, and the ER clearance from the mitotic spindle was observed both in control cells and LAT1-knockdown cells (Fig. S2*F*), suggesting that LAT1 knockdown-induced mitotic delay is not caused by the abnormal accumulation of ER on the mitotic spindle. Collectively, these results suggest that Golgi-localized LAT1 contributes to Golgi unlinking during the mitotic entry and subsequently promotes centrosome maturation.

## Discussion

In this study, we demonstrated that the large neutral amino acid transporter LAT1 is important for proper mitotic progression. LAT1 knockdown shows the spindle orientation defects along with the improper cell cortex localization of the force generator complex protein NuMA. Interestingly, the activity of the amino acid transport is dispensable for the regulation of spindle orientation and mitotic progression. Of note, LAT1 is localized at the Golgi region and regulates Golgi unlinking during the mitotic entry, which is required for the localization of centrosomal Aurora A. These results highlight a novel function of the amino acid transporter LAT1 in mitosis.

Amino acid uptake contributes to the proper protein abundance within a cell, thereby controlling various cellular functions (42). LAT1 is a sodium- and pH-independent large neutral amino acid transporter (43). Considering the importance of LAT1 for protein synthesis, we hypothesized that LAT1 knockdown would result in various defects within the whole mitotic process; however, LAT1 knockdown did not affect mitotic entry but caused a delay in mitotic progression (Figs. 1 and 3). Furthermore, LAT1 knockdown activated SAC and specifically delayed the onset of anaphase along with spindle misorientation (Figs. 1, 3 and 4). Interestingly, JPH203 treatment did not affect mitotic progression in HeLa S3 and MIA PaCa-2 cells (Figs. 2, S1). A LAT1 mutant that lacked amino acid uptake ability could support mitotic progression similar to the wild-type LAT1 (Figs. 2, 4). Collectively, these results suggest that LAT1 *per se* contributes to proper SAC satisfaction during metaphase in a transport activity-independent manner.

The heterodimerization of LAT1 with CD98 is not essential for amino acid uptake at the plasma membrane, but it is important for the trafficking of LAT1 from the perinuclear region to the plasma membrane (17). Indeed, the plasma membrane localization of LAT1 was poorly observed in the CD98 knockdown-interphase and -mitotic cells (Fig. 5, *D* and *E*). Interestingly, the drastic change in LAT1 localization by CD98 knockdown did not affect mitotic progression and spindle orientation (Fig. 5*F*). This suggests the possibility that intracellular LAT1 regulates proper mitotic progression. LAT1 is presumed to be synthesized in the ER and transported to the plasma membrane through the Golgi region. Indeed, endogenous LAT1 was observed at the Golgi and ER (Fig. 6*A*). Unlinking the interconnection between Golgi stacks during late G2 and prophase is important for mitotic entry and subsequent spindle formation through centrosomal Aurora A-mediated centrosome maturation (9, 10). Although the Golgi structure is spread out in prophase cells (Fig. S2*C*), LAT1 knockdown repressed it (Fig. 6, *B* and *C*). LAT1 knockdown also repressed Aurora A recruitment to the centrosomes (Fig. 6, *D* and *E*). Similar results were observed following the inhibition of JNK signaling (Fig. 6, *F* and *G*), in which JNK phosphorylates GRASP65 during the late G2 phase, a Golgi membrane-tethering protein, and promotes Golgi unlinking (10). After prophase, further phosphorylation of GRASP65 at T220/T224 by CDK1 leads to cisternal unstacking along with GRASP55 phosphorylation, and several proteins promote vesiculation of the Golgi membranes. Alanine mutations of T220/T224 in GRASP65 inhibit the Golgi fragmentation and induce large Golgi punctae in metaphase cells (44). Since LAT1 knockdown did not repress Golgi fragmentation in metaphase cells (Fig. S2*E*), LAT1 may specifically promote Golgi unlinking during the mitotic entry. These results suggest a possibility that Golgi membrane-localized LAT1 promotes Golgi unlinking and subsequent centrosome maturation.

Time-lapse imaging analysis and immunofluorescence analysis clearly showed that LAT1-knockdown cells exhibited spindle misorientation (Figs. 3 and 4). Re-expression of the LAT1 mutant lacking transport activity mitigated the defect in spindle orientation caused by LAT1 knockdown, suggesting that LAT1 maintains spindle orientation in a transport activity-independent manner. Since centrosomal Aurora A is important for spindle orientation (45), LAT1-mediated centrosome maturation *via* Golgi unlinking likely maintains the proper spindle orientation by promoting Aurora A activation at the centrosomes. LAT1 also regulates the confined lateral cortex localization of NuMA (Fig. 4). The NuMA/LGN/Gαi complex anchors the minus end-directed dynein–dynactin complex, thereby generating a proper pulling force on the mitotic spindle toward the lateral cortex (33). Previous reports showed that NuMA phosphorylation at Ser1969 by Aurora A enhances its mobility and prevents its accumulation at the polar region, thereby promoting lateral cortex localization (4, 46). In contrast, unconfined localization of NuMA was frequently observed rather than loss of cortical localization in the present study. We found that LAT1 knockdown reduced centrosomal localization of active Aurora A (Fig. 6*D*). Given that delocalized Aurora A from the centrosomes would phosphorylate lateral cortex–localized NuMA, phosphorylated NuMA may be released from the lateral cortex and result in delocalization throughout the cortex. Moreover, CDK1-mediated phosphorylation of NuMA at Thr2055 contributes to the confined localization of NuMA (47); therefore, the LAT1 knockdown-induced unconfined localization of NuMA by an unknown mechanism might also contribute to the spindle misorientation due to the dysregulated pulling force on the mitotic spindle.

A critical feature of the LAT1 transporter is strongly elevated expression in various cancer cells (48), suggesting that the elevated uptake of amino acids is important for cancer progression (49). What is the significance of highly expressed LAT1-mediated support of mitotic progression in cancer cells? Cancer cells undergo uncontrolled mitosis and proliferation. Since strong SAC activation caused by mitotic defects leads to mitotic cell death and proliferation inhibition (50), proper mitotic progression is required by cancer cells to an extent. Various mitotic regulators are highly expressed in cancer cells and support their division (51, 52). High expression of these regulators often correlates with a poor prognosis in patients with cancer. Therefore, highly expressed LAT1-mediated proper mitotic progression may be involved in the progression of cancer by adapting cancer cells to uncontroled mitosis and increasing the intratumoral heterogeneity.

In conclusion, we revealed a transport activity-independent function of LAT1 that supports mitotic progression *via* the promotion of Golgi unlinking and subsequent centrosome maturation. Inhibitors of LAT1 transport activity, such as JPH203 and OKY-034, have been previously developed (53) and have undergone clinical trials in patients with pancreatic or biliary tract cancer (54). Since only compounds targeting its transport activity have been explored, the strategy for the repression of LAT1 protein expression might lead to the efficient inhibition of cancer progression by limiting protein synthesis and inducing mitotic arrest. It has been recently reported that thalidomide derivatives suppress the protein expression of membrane proteins, including LAT1, *via* the dysregulation of the chaperone function of HSP90, thereby causing the efficient suppression of cancer proliferation (55). Further studies are required to reveal the mechanism of LAT1-mediated regulation of cell division and its clinical significance.

## Experimental procedures

### Cells

HeLa S3 (Japanese Collection of Research Bioresources), MIA PaCa-2 (RIKEN BRC), and Lenti-X 293T (Clontech Laboratories) cells were cultured in Dulbecco’s modified Eagle’s medium (DMEM) containing 5% fetal bovine serum (FBS) with 20 mM HEPES–NaOH (pH 7.4) at 37 °C in 5% CO_2_. To examine the effect of JPH203 treatment on the proliferation and mitotic progression of MIA PaCa-2 cells, the amino acid concentration of the LAT1 substrate in the culture medium was reduced closer to those of physiological levels (0.3 × DMEM), as previously reported (49). The medium was obtained by combining two volumes of DMEM lacking the five essential amino acids Leu, Ile, Met, Phe, and Trp (D9800-13, United States Biological, Swampscott) with one volume of regular DMEM.

### Plasmids

pDONR221_SLC7A5 was a gift from RESOLUTE Consortium and Giulio Superti-Furga (Addgene plasmid # 132197; http://n2t.net/addgene:132,197; RRID: Addgene_132197). The siRNA-resistant LAT1 construct was created by PCR-mediated introduction of a silent mutation into the siLAT1#2 target site with (sense) 5′-GTTTACCTGCGTCATGACACTGCTGTATGCC-3′ and (antisense) 5′-ATGACGCAGGTAAACACCAGGCTGGGCACTG-3′, and the hemagglutinin (HA)-tag was inserted at the C-terminus of the LAT1 cDNA (pDONR221_LAT1-WT). The mutant LAT1 with the Trp257 to Ala substitution (W257A) was generated (pDONR221_LAT1-W257A) by inverse PCR from the template pDONR221_LAT1-WT plasmid with (sense) 5′-GCCAACTATCTGAATTTTGTGACAGAGGAGATG-3′ and (antisense) 5′-GCCGCCGTAGGCGAACAG-3′. pDONR221_LAT1-WT and pDONR221_LAT1-W257A were recombined with pLIX_402 (gifted by David Root, plasmid 41,394; Addgene) (56) lentiviral plasmids using the Gateway LR reaction according to the manufacturer’s instruction (pLIX_402_LAT1-WT, LAT1-W257A) (Thermo Fisher Scientific).

### Establishment of LAT1-inducible cell lines *via* lentiviral transduction

To establish the Dox-inducible cell lines of the C-terminal HA-tagged wild-type LAT1 and mutant LAT1 with Trp257 to Ala substitution, Lenti-X 293T cells were cotransfected with 1.2 μg of pLIX_402 vector harboring either construct, 0.8 μg of pCAG-HIVgp, and 0.8 μg of pCMV-VSV-G-RSV-Rev using Lipofectamine 2000 (Thermo Fisher Scientific) in a 35-mm dish. After overnight incubation, the medium was changed to a fresh medium containing 10 μM Forskolin. The virus-containing medium was harvested 1 day after transfection and passed through a 0.45-μm filter. The HeLa S3 cells were infected with the virus-containing medium in the presence of 80 μg/ml polybrene (MilliporeSigma) and selected with 2 μg/ml puromycin (HeLa S3/LAT1-WT or LAT1-W257A) (StressMarq Biosciences).

### siRNA

HeLa S3 and MIA PaCa-2 cells were transfected with five or 10 pmol/well of siRNA in a 24-well plate or 1.25 pmol/well of siRNA in a 96-well plate using the Lipofectamine 2000 reagent. siLAT1#1 (5′-TGACCAACCTGGCCTACTT-3′) was synthesized by MilliporeSigma, and siLAT1#2 (SASI_Hs01_00103508, 5′-GTGCCGTCCCTCGTGTTCA-3′) was purchased from MilliporeSigma. siCD98#1 (J-003542-09, 5′-GGACCTTACTCCCAACTAC-3′) and siCD98#2 (J-003542-10, 5′-GAATGAGCGTTTTCTGGTA-3′) were purchased from Horizon Discovery (Cambridge, UK).

### Antibodies

The following primary antibodies were used for immunofluorescence (IF) and immunoblotting (IB): rat monoclonal anti-α-tubulin (IF, 1:800; IB, 1:4000; MCA78G, Bio-Rad), rabbit polyclonal anti-LAT1 (IB, 1:4000; #5347, Cell Signaling Technology), rabbit polyclonal anti-LAT1 (IF, 1:200; KE026, Trans Genic Inc), mouse monoclonal anti-phospho-Hisotone H3 (pS10) (IF, 1:400; #9706, Cell Signaling Technology), mouse monoclonal anti-HA-tag (IF, 1:500; IB, 1:1000; M180-3, Medical and Biological Laboratories), mouse monoclonal anti-γ-tubulin (IF, 1:500; GTU-88, MilliporeSigma), mouse monoclonal anti-NuMA (IF, 1:200; sc-365532, Santa Cruz Biotechnology), mouse monoclonal anti-CD98 (IB, 1:1000; sc-376815, Santa Cruz Biotechnology), sheep polyclonal anti-TGN46 (IF, 1:1000; AHP500GT, Bio-rad), mouse monoclonal anti-Calnxin (IF, 1:400; sc-46669, Santa Cruz Biotechnology), mouse monoclonal anti-Aurora A (IF, 1:400; #610938, BD Biosciences), rabbit monoclonal anti-phosho-Aurora A (pT288) (IF, 1:100; #30792, Cell Signaling Technology), rabbit monoclonal anti-GM130 (IF, 1:100; #12480, Cell Signaling Technology), and mouse monoclonal anti-cyclin B1 (IF, 1:50; sc-245, Santa Cruz Biotechnology) antibodies. We validated the antibodies for IF based on the appropriate fluorescence signal of the protein at the previously reported localization, or by observing a decrease in the fluorescence signal following the knockdown of the protein. We validated the antibodies for IB based on the correct band size of the protein or the decrease in the level of the protein band following knockdown. For IF, Alexa Fluor 488-, 555-labeled donkey anti-mouse, anti-rat, and anti-sheep (1:800; Life Technologies) IgG antibodies were used. For IB, horseradish peroxidase-conjugated anti-mouse (1:8000; 715-035-151), anti-mouse IgG Fcr fragment specific (heavy chain) (1:8000; 115-035-071), anti-rabbit (1:8000; 711-035-152), and anti-rat (1:8000; 712-035-153) IgG antibodies were purchased from Jackson ImmunoResearch (West Grove).

### Cell cycle synchronization

To analyze the effects of knockdown or knockdown-rescue of LAT1 on mitotic progression, the cells were arrested at the G2/M border by treating the cells with the CDK1 inhibitor RO-3306 at 6 μM for 20 h. To release cells from G2/M arrest, the cells were washed with prewarmed PBS supplemented with Ca^2+^ and Mg^2+^ [PBS(+)] four times on a water bath at 37 °C and incubated in the prewarmed medium. Subsequent incubation time was 75 min for the HeLa S3 cells or 90 min for the LAT1-WT and LAT1-W257A cells. The cells were then fixed with 2% formaldehyde in PBS(−) for 20 min at room temperature. The fixed cells were stained for α-tubulin and DNA, and the mitotic cells were classified into four categories: prophase/prometaphase (P/PM), metaphase (M), anaphase/telophase (A/T), and cytokinesis (C). The percentage of each category was then calculated. In addition, to examine the percentage of synchronized cells that could enter mitosis, we calculated the percentage of mitotic cells in each experiment. Alternatively, the HeLa S3 cells were pretreated with 4 mM thymidine (MilliporeSigma) for 20 h. After a 9-h release from thymidine treatment, we performed time-lapse imaging. To analyze the degree of Golgi unlinking or the fluorescence intensity of Aurora A, cells were fixed after 9 to 11 h of release from thymidine synchronization. S-trityl-L-cysteine (STLC; MilliporeSigma), which inhibits the Eg5 kinesin motor protein, was used for cell cycle synchronization in the mitosis by incubation for 16 h. Mitotic cells were collected by mitotic shake-off for use in western blotting and immunoprecipitation.

### Immunofluorescence microscopy

For α-tubulin, phospho-Histone H3 (pS10), LAT1, TGN46, GM130, or calnexin staining, formaldehyde-fixed cells were permeabilized and blocked with PBS(−) containing 0.1% saponin and 3% BSA for 30 min, incubated with the primary antibody for 1 h, and subsequently with the secondary antibody for 1 h along with 1 μM Hoechst 33342 for DNA staining. For γ-tubulin, LAT1, TGN46, Aurora A, phospho-Aurora A (pT288), or cyclin B1 staining, cells were fixed with 100% MeOH for 5 min at − 30 °C and stained as described above. For NuMA staining, the cells were fixed with 100% MeOH for 5 min at − 30 °C, permeabilized with PBS(−) containing 0.2% Triton X-100 for 10 min at room temperature, blocked with PBS(−) containing 0.1% Triton X-100 and 3% BSA for 30 min, and incubated with the primary and secondary antibodies as described above. The fluorescence images were obtained using an IX-83 fluorescence microscope (Olympus) equipped with a × 20/0.45 NA or a × 60/1.42 NA oil-immersion objective lens (Olympus). The optical system included a U-FUNA filter cube (360–370 nm excitation, 420–460 nm emission), a U-FBNA cube (470–495 nm excitation, 510–550 nm emission), and a U-FRFP cube (535–555 nm excitation, 570–625 nm emission) to observe the Hoechst 33342, Alexa Fluor 488, and Alexa Fluor 555 fluorescence, respectively. A MAICO MEMS confocal unit C15890 (Hamamatsu Photonics) was used to capture the z-stack images of mitotic cells in Fig. 4, *A*–*C* or analyze the endogenous LAT1 localization in Figs. 6*A* and S2*B*. The captured images were edited using ImageJ (NIH), Photoshop CC, and Illustrator CC software (Adobe). To analyze the spindle orientation in Fig. 4, *A* and *B*, we calculated the difference between the heights of the two centrosomes within a cell. We classified the plasma membrane localization of NuMA into three groups in Fig. 4*D*: one or both sides of the lateral cortex (lateral), unobserved (none), or at the lateral cortex plus the center cortex (lateral + center). To analyze the degree of Golgi unlinking per cell in Fig. 6, we stained TGN46, captured z-stack images, and measured the number of “Golgi object” using ImageJ software, as described previously (14). We projected z-stack images into a single layer by maximum intensity projection, applied “Subtract Background” using a rolling ball radius of 40 pixels, manually applied a threshold, and counted objects above the threshold fluorescent as ‘Golgi object’ using the “Analyse Particle” function with a size range of five to infinity, as indicated in Fig. S2*D*. The mean fluorescence intensity of Aurora or phospho-Aurora A in Fig. 6 was calculated as the difference between a circular area of interest and an identically sized neighboring area using the ImageJ software, as described previously (25).

### Time-lapse imaging

Time-lapse imaging was performed as previously described (57). HeLa S3 or MIA PaCa-2 cells were transfected with siRNAs or treated with JPH203 as described in the figure legends. The cells were then treated with 0.1 μM Hoechst 33342, and time-lapse imaging was performed using a high-content imaging system (Operetta, PerkinElmer Life Sciences) at 37 °C in 5% CO_2_.

### Western blotting

Western blotting was performed as previously described (58). Briefly, the cells were lysed in SDS sample buffer containing protease inhibitors [10 μg/ml aprotinin (Fujifilm Wako Pure Chemicals), 4 μg/ml pepstatin A (Peptide Institute, Inc), 10 μg/ml leupeptin (Nacalai Tesque), 2.5 mM EGTA-KOH (Sigma), and 1 mM phenylmethylsulfonyl fluoride (PMSF, Nacalai Tesque)] and denatured at 70 °C for 10 min. To detect the dimerized form of LAT1 and CD98, cells were lysed in SDS sample buffer with or without 2-ME and denatured at 70 °C for 10 min, as previously described (36). Whole-cell lysates were subjected to SDS–PAGE and electrotransferred onto polyvinylidene difluoride membranes (PVDF; Pall Corporation). To discriminate the bands of endogenous LAT1 and Dox-induced HA-tagged LAT1 (LAT1-HA) in Fig. 2*D*, we used polyacrylamide gels containing 6 M urea. Blocking to minimize nonspecific interactions was done with Blocking One (Nacalai Tesque) at room temperature for 30 min. The membranes were then incubated with antibodies diluted with Tween 20-containing Tris-buffered saline with 5% Blocking One. Clarity (Bio-Rad) was used as the chemiluminescence substrate, and a ChemiDoc XRSplus image analyzer (Bio-Rad) was used for chemiluminescence detection and band intensity analysis.

### Amino acid uptake assay

The amino acid uptake capacity of cells was assessed using the Amino Acid Uptake Assay Kit (#UP04-12, DOJINDO Laboratories) according to the manufacturer’s instructions. Briefly, HeLa S3 cells were cultured in regular DMEM as mentioned above and seeded in a 96-well plate (655,180; Greiner Bio-One). The cells were transfected with siRNAs or treated with JPH203 for 2 days. The cells were then washed three times with Hanks’ Balanced Salt Solution containing Ca^2+^ and Mg^2+^ [HBSS(+)], incubated with BPA uptake solution for 5 min at 37 °C in 5% CO_2_, washed again with HBSS(+) three times, and incubated with a working solution containing the BPA probe for 5 min at 37 °C in 5% CO_2_. Fluorescence images were obtained using a high-content imaging system (Operetta). The mean fluorescence intensity of the BPA–probe fluorescent compound in the cells was determined using ImageJ software.

### Immunoprecipitation

Immunoprecipitation of HA-tagged LAT1-WT from the mitotic cells was performed. HeLa S3 cells expressing inducible LAT-WT were arrested at mitosis by treatment with 5 μM STLC. After mitotic shake-off, the collected mitotic cells were solubilized at 4 °C for 10 min in 1% Triton lysis buffer (25 mM HEPES–NaOH, 2 mM EDTA-NaOH, 5% glycerol, 50 mM NaF, 2 μg/ml aprotinin, 0.8 μg/ml pepstatin A, 2 μg/ml leupeptin, 2 mM PMSF, 20 mM β-glycerophosphate, 10 mM Na_3_VO_4_, and 1% Triton X-100), followed by centrifugation to remove insoluble materials. The resulting lysates were subjected to immunoprecipitation using protein G-Sepharose beads (GE Healthcare) precoated with mouse IgG (sc-2025; Santa Cruz Biotechnology) or anti-HA (M180-3, Medical and Biological Laboratories) antibodies. After incubation for 3 h at 4 °C, the beads were washed three times with Triton lysis buffer containing 0.01% Triton X-100, and the immunoprecipitants were subjected to western blotting analysis.

### Proliferation assay

The proliferation of MIA PaCa-2 cells was determined using a Cell Counting Kit-8 (Dojindo) according to the manufacturer’s instructions, as previously described (59). MIA PaCa-2 cells were cultured in 0.3 × DMEM as mentioned above, seeded in 96-well plates (3 × 10^3^ cells/well), and treated with 3 to 100 μM of JPH203, an inhibitor of the transport activity of LAT1, for 3 days. As a solvent control, cells were cultured in the presence of 1% DMSO. Based on the absorbance (450 nm) of the reduced 2-(2-methoxy-4-nitrophenyl)-3-(4-nitrophenyl)-5-(2,4-disulfophenyl)-2H-tetrazolium monosodium salt (WST-8), the number of cells was evaluated. The absorbance of the control cells treated with DMSO was set to 1, and the ratio of the absorbance of the inhibitor-treated cells to that of the control cells was calculated.

### Statistics

Statistical differences between two datasets were analyzed using Student’s *t* test after analysis of variance by F-test. Statistical differences among more than two datasets were analyzed using one-way ANOVA or two-way ANOVA with Tukey’s *post hoc* test Dunnett’s *post hoc* test, or Welch’s ANOVA with Games–Howell *post hoc* test, depending on their variance that was analyzed using Bartlett’s test. Statistical analysis was performed using EZR software (v.1.55; Saitama Medical Center, Jichi Medical University) (60) and R software (v.4.1.2; R Foundation for Statistical Computing).

## Data availability

The data used to support the findings of the study are available from the corresponding author upon reasonable request.

## Supporting information

This article contains supporting information.

## Conflict of interest

The authors declare that they have no conflicts of interest with the contents of this article.
