# Supplementary material for: LAT1 supports mitotic progression through Golgi unlinking in an amino acid transport activity-independent manner
Source: J Biol Chem. 2024 Sep 11;300(10):107761. doi: 10.1016/j.jbc.2024.107761 (PMC11490712; doi:10.1016/j.jbc.2024.107761)
Supplement: Supporting information [file mmc1.docx]

**LAT1 supports mitotic progression through Golgi unlinking in an amino acid transport activity-independent manner**

**Sakura Yanagida, Ryuzaburo Yuki^*^, Youhei Saito, and Yuji Nakayama^*^**

**Supporting information**

**Fig. S1. JPH203 treatment does not affect mitotic progression in MIA PaCa-2 cells.**

(**A, B**) MIA PaCa-2 cells were cultured in DMEM with reduced essential amino acids (0.3 × DMEM, see “Experimental procedures”). (**A**) Cells were treated with 3–100 µM JPH203 for 72 h, and then cell viability was determined using a WST-8 assay. The relative absorbance ratios to the solvent control are plotted as the mean ± SD calculated from three independent experiments. The IC50 was calculated in each experiment and is shown as the mean ± SD. (**B**) Cells were treated with 50 μM JPH203 for 48 h, and the cells were monitored for a further 12 h by time-lapse imaging with 0.1 µM Hoechst 33342. The graphs are shown as indicated in Fig. 2C.

**Fig. S2. Staining of endogenous LAT1, and LAT1 knockdown effect on the Golgi structure and ER distribution in mitotic cells.**

(**A**) HeLa S3 cells were fixed with MeOH or formaldehyde, and stained with LAT1 (green) and DNA (red). Scale bar, 20 µm. (**B**) HeLa S3 cells were transfected with siControl or siLAT1#1. At 48 h after transfection, cells were fixed with formaldehyde and stained for LAT1 (green) and DNA (red). Representative images are shown. Scale bar, 10 µm. (**C**) HeLa S3 cells were fixed with MeOH and stained for TGN46 (gray or green), DNA (red), and cyclin B1(magenta). We determined Cyclin B1-negative cells as G1/S, cells showing strong Cyclin B1 expression in cytosol but not in nucleus as Early G2, or cells having condensed chromatin as Prophase. Scale bar, 10 µm. (**D**) Representative images being processed using Image J in Fig. 6B are shown (siControl) (see “Experimental procedures”). Scale bar, 10 µm. (**E**) HeLa S3 cells were transfected with siControl, siLAT1#1, or siLAT1#2. At 48 h after transfection, cells were fixed with MeOH and stained for TGN46 (gray), GM130 (gray), and DNA (red). Representative images are shown. Scale bar, 10 µm. (**F**) HeLa S3 cells were transfected with siC ontrol, siLAT1#1, or siLAT1#2. At 48 h after transfection, cells were fixed with formaldehyde and stained for Calnexin (green), and α-tubulin (red). Representative z-stack images are shown. Scale bar, 10 µm.

**Fig. S3. Full-length blots.**
