# Supplementary figures and images for: LAT1 supports mitotic progression through Golgi unlinking in an amino acid transport activity-independent manner
Source: J Biol Chem. 2024 Sep 11;300(10):107761. doi: 10.1016/j.jbc.2024.107761 (PMC11490712; doi:10.1016/j.jbc.2024.107761)

**A**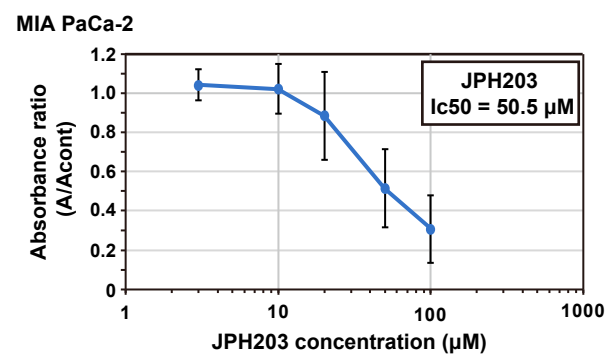**B**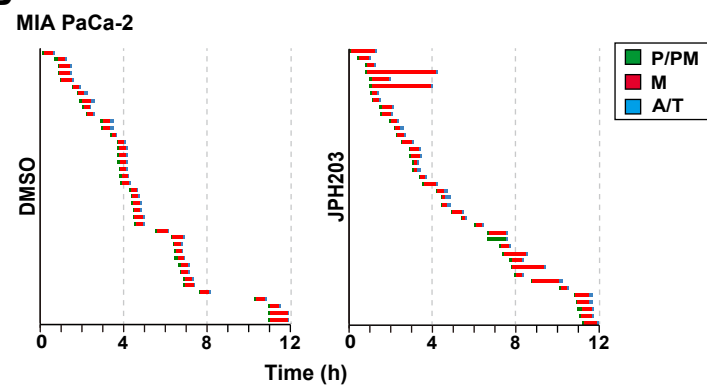

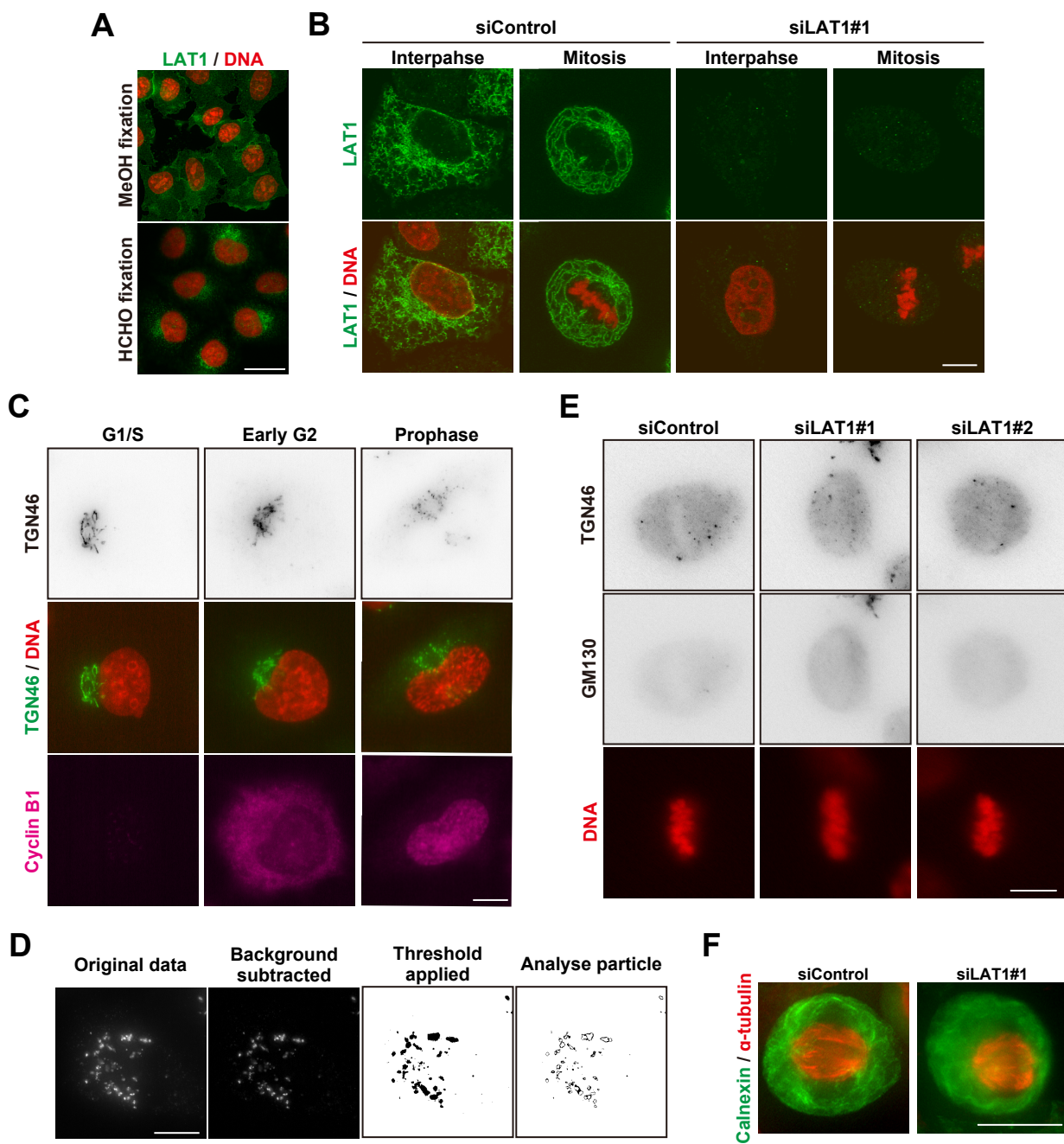

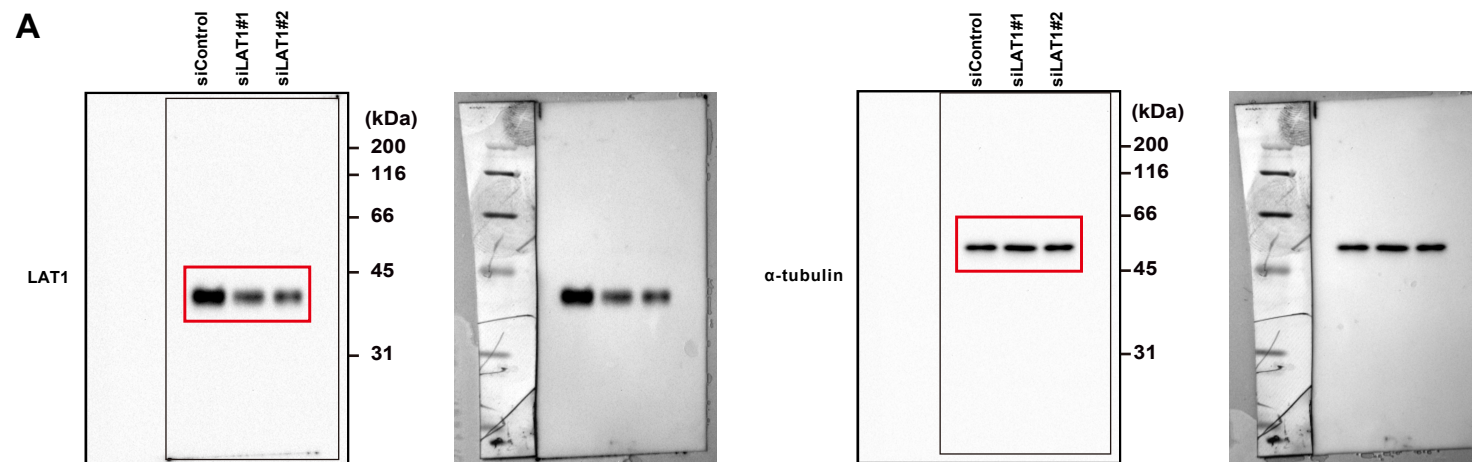

**B**

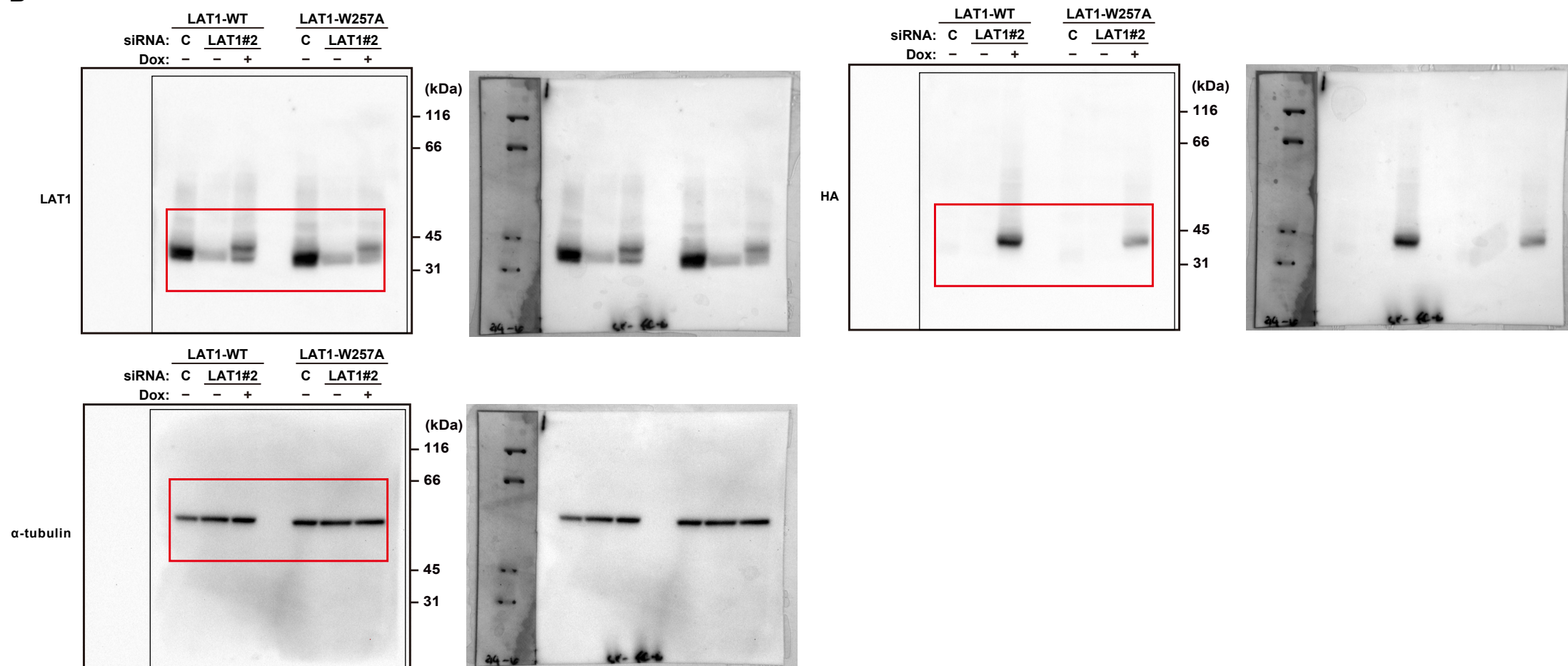

**C**

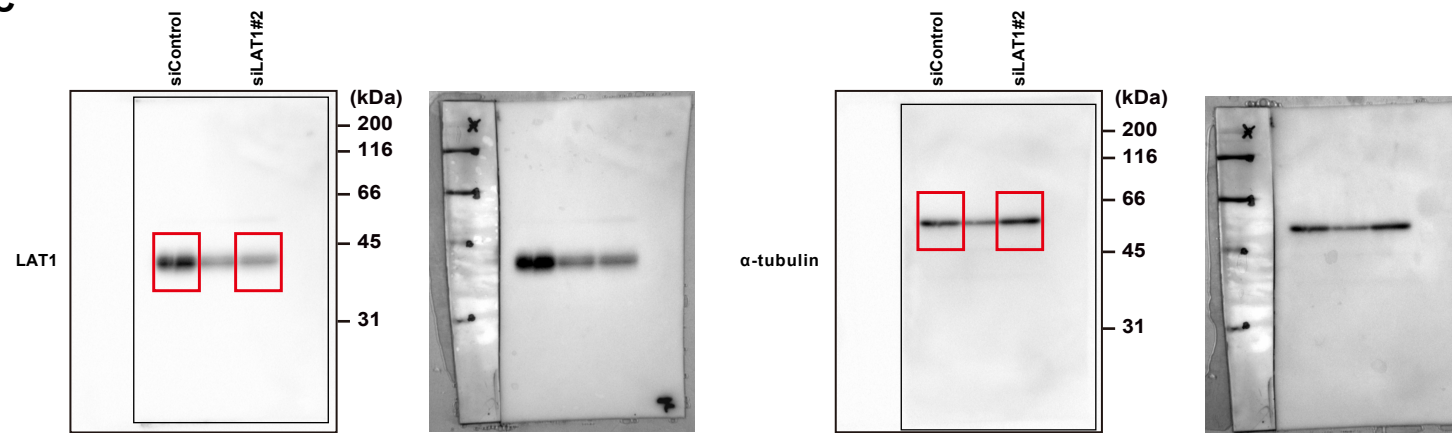

**D**

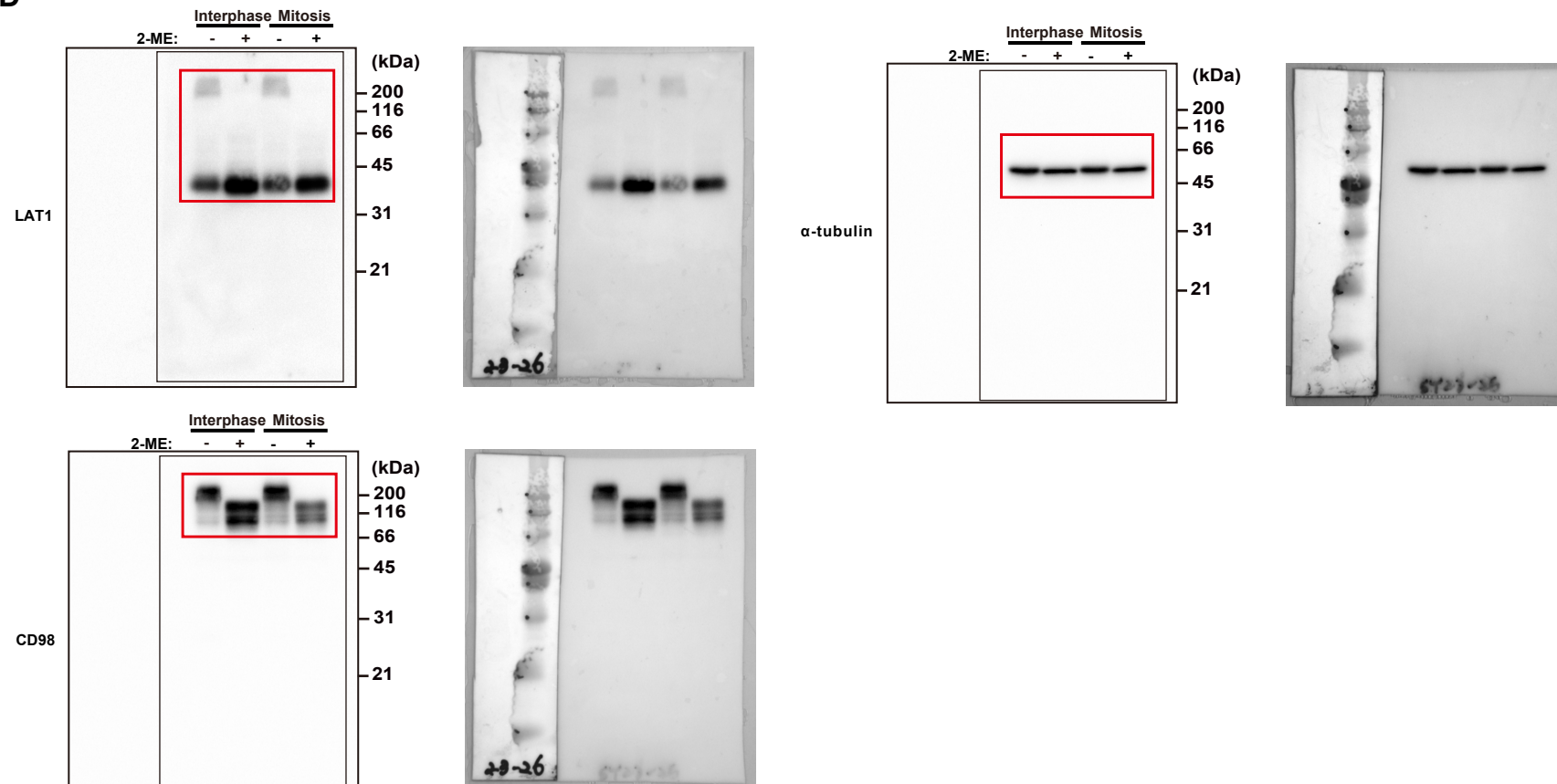

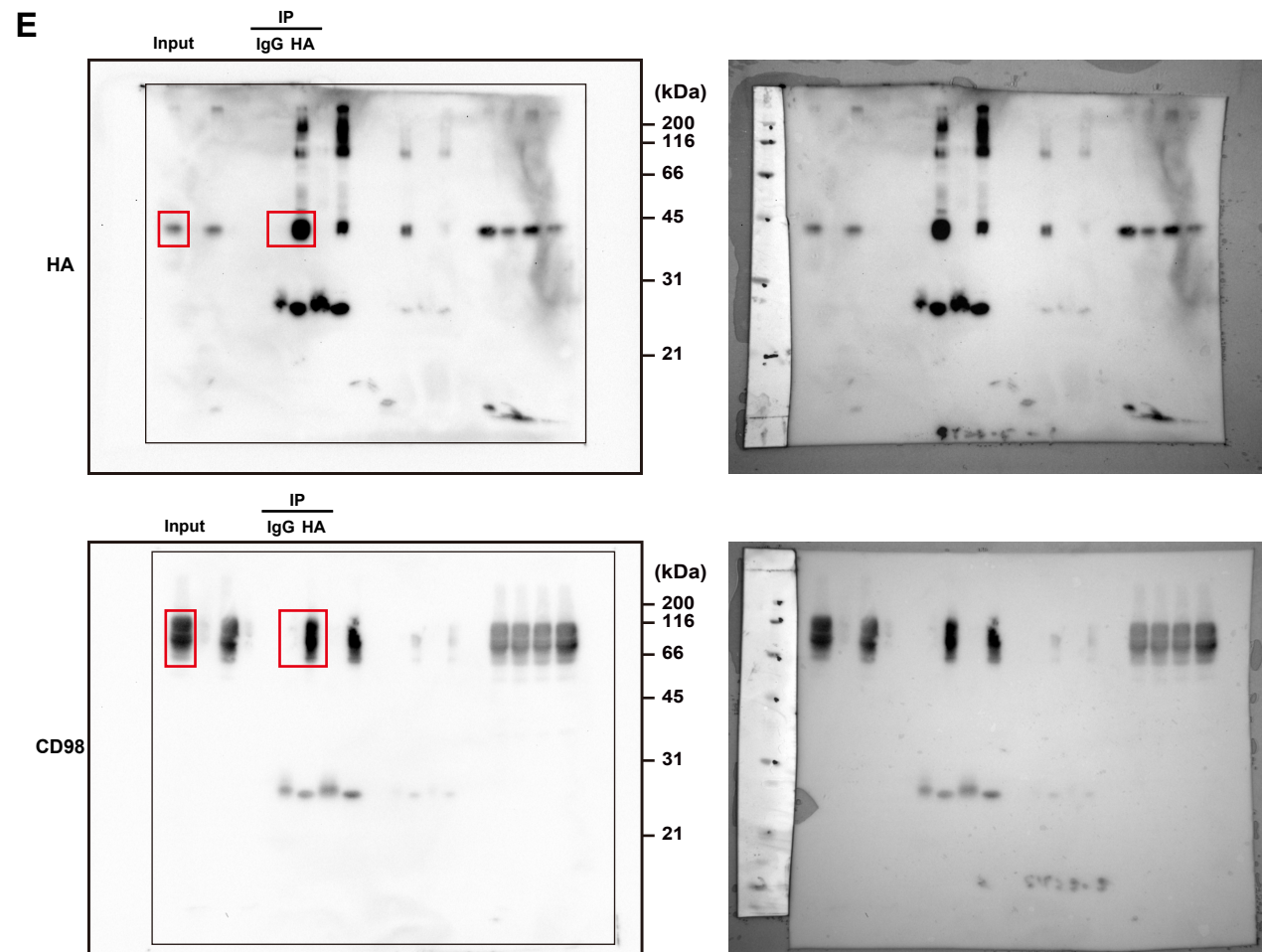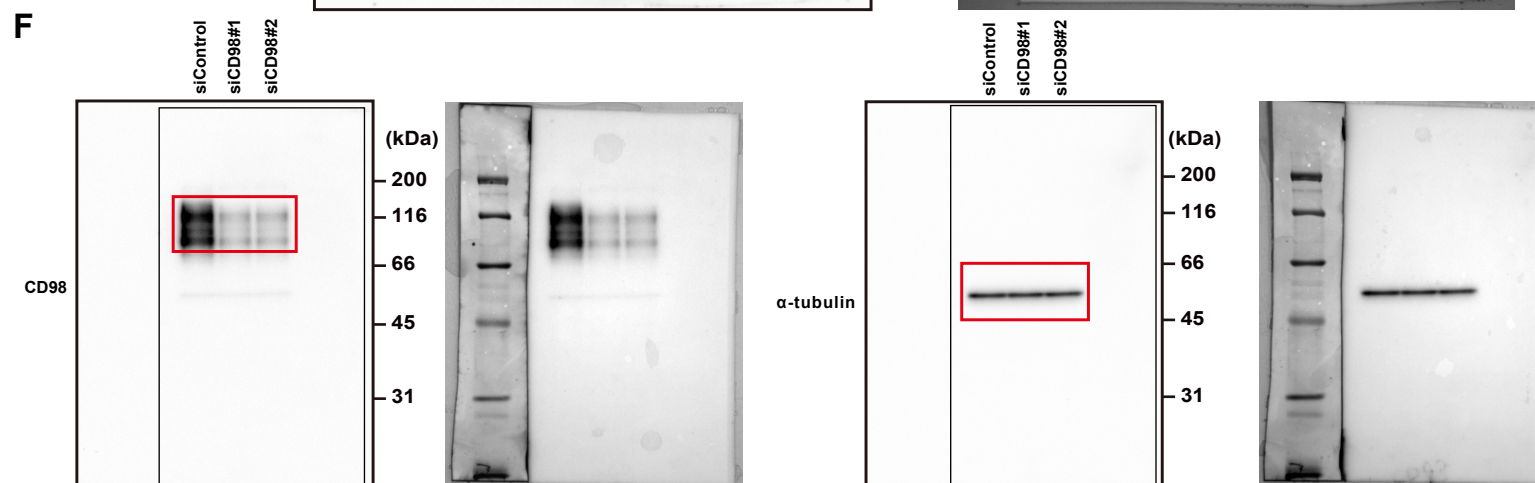

Supplementary figure 3-3

Supplement: Supplemental Figures S1–S3 [file mmc2.pdf]
